# Comparison of Sleep Quality before and after Chemotherapy in Locally Advanced Nonsmall Cell Lung Cancer Patients: A Prospective Study

**DOI:** 10.1155/2020/8235238

**Published:** 2020-06-30

**Authors:** N. Belloumi, S. Maalej Bellaj, I. Bachouche, F. Chermiti Ben Abdallah, S. Fenniche

**Affiliations:** ^1^Pulmonology Department Pavilion 4 Abderrahman Mami Hospital Ariana, Tunisia; ^2^Pulmonology Department Pavilion D Abderrahman Mami Hospital Ariana, Tunisia; ^3^University of Tunis El Manar, Faculty of Medicine of Tunis, Tunisia

## Abstract

**Background:**

Preservation of sleep quality is a modifiable and treatable factor, which may enhance the patient's adherence to other supportive and palliative care procedures. The outcome of sleep disturbances in lung cancer patients before and after treatment aren't reported. The aim of this study was to investigate changes in sleep quality before and after chemotherapy in locally advanced or metastatic NSCLC patients.

**Methods:**

It was a prospective study including 64 patients with stage III or IV nonsmall-cell lung cancer. Patients answered the Tunisian dialectal version of the following questionnaires: PSQI and QLQ-C30 in order to evaluate, respectively, the sleep quality and the quality of life. The assessments took place before chemotherapy and then repeated after the chemotherapy course was over.

**Results:**

The mean age was 62.9 years. All patients were active smokers. Before chemotherapy, there were 10 patients (15%) with poor sleep quality. The most frequent complaints were daytime sleepiness (70%) and nocturnal arousals (100%). After chemotherapy, the mean PSQI score increased from 2.9 to 5.4, and 45% of all patients had poor sleep quality. Most frequent complaints were the extension of sleep latency (69%), daytime sleepiness (98%), and nocturnal arousals (100%). Predicting factors of sleep disturbance according to statistical univariate analysis were delayed diagnosis confirmation (*p* = 0.05), delayed treatment onset (*p* < 10^−3^), depressive mood (*p* = 0.001), and anxious mood (*p* = 0.001). Multivariate analysis had shown a significant and independent correlation between sleep quality and shortened diagnosis and treatment delays. Sociodemographic parameters, clinical parameters, and factors related to treatment procedure had no correlation with sleep quality.

**Conclusions:**

Our study demonstrates the persistence and potential intensity worsening of sleep disturbances in advanced stage nonsmall-cell lung cancer patients. We, hereby, reported a statistical correlation between sleep quality and quality of life in our patients.

## 1. Background

According to the Global Cancer Observatory, worldwide incidence was 2.09 million cases in 2018. It is also responsible of 1.76 million deaths per year. Lung cancer represents 11.6% of all malignant tumor incidence and makes 18.4% of mortality of all cancers. It has so, the highest mortality rate in men and the second-highest mortality rate in women [1, 2]. In Tunisia, tobacco smoking is still rising. Lung cancer incidence in men is 63.5 cases/100000 in the period 2004-2006. Incidence in women is 5.1 cases/100000 [3]. Diagnosis announcement is followed therefore by a psychological impact with variable intensity and various manifestations. Chemotherapy is proposed in stage IV of the disease if there are no contraindications. Its impact on the general status, quality of life, and sleep quality is scarcely reported [4–6]. These interactions between burdensome therapeutic protocols and quality of life or sleep were reported in other cancer types [7–9]. Preservation of sleep quality is a major palliative care goal increasingly mentioned in advanced cancer management. Nevertheless, sleep quality deterioration before and after chemotherapy has not been studied. The aim of this study was to report various sleep disturbances in advanced-stage lung cancer patients and their outcome after chemotherapy and/or radiotherapy. We aimed also to assess the correlation between sleep quality and quality of life.

## 2. Methods

### 2.1. Design

This was a single-arm prospective cohort study conducted in the pulmonology department Pavilion 4 in Ariana Hospital, Tunisia. The follow-up period was the duration of the chemotherapy course.

### 2.2. Patients

We included all NSCLC patients in stage III to IV of the disease according to the 8th edition of TNM classification [10].

*Inclusion criteria* were confirmed NSCLC with histological proof, stage III or IV of the disease, prescription of a chemotherapy course, written informed consent to receive chemotherapy course, and written informed consent to participate in the study.

*Noninclusion criteria* were known sleep disturbance prior to lung cancer diagnosis, pharmacological treatment interfering with sleep quality, severe pain with intensity greater than 6/10 in visual analogical scale (VAS), and leading to the prescription of oral opioïd therapy.

*Exclusion criteria* were the death of the patient during the chemotherapy course, worsening of the general status leading to a medical decision to stop the chemotherapy, nonadherence of the patient to chemotherapy whatever was the reason, withdrawal of consent to participate in the study, and starting a prescription of any treatment interfering with sleep quality: morphine, neuroleptic, or antiepileptic treatment.

### 2.3. Methods and Assessment Tools

The inclusion period lasted from February to August 2018. We included consenting patients who fulfilled the inclusion criteria. After announcing the diagnosis and the prognosis of the disease, we planned an appointment with the patient before starting the chemotherapy protocol. In this meeting, we made our first evaluation via questionnaires of the quality of life and sleep quality. We gathered also sociodemographic and clinical data. After the chemotherapy course, if there were no exclusion criteria, a second evaluation with the same methods was done. Side effects of chemotherapy administration were classified as mentioned in the “Common Terminology Criteria for Adverse Events” [11].

#### 2.3.1. Sleep Quality Assessment

Pittsburgh Sleep Quality Index (PSQI) was used. It contains nineteen questions about seven dimensions of sleep disturbance patterns: global subjective quality of sleep—sleep latency—sleep duration—sleeping efficiency—sleep and wake disturbances—use of sleep medication—daytime dysfunction or sleepiness [12]. Every dimension is evaluated from 0 to 3 according to the patient's answers. The score reflects the disturbance severity. The seven dimensions scores were combined to give a global score ranging from 0 to 21, with greater scores indicating poorer sleep quality. When the PSQI score is greater than 5, sleep quality is considered as poor. The PSQI was validated for cancer patients [13]. We used an Arabic Tunisian dialectal version of PSQI, which is not validated yet, to be easily answered by patients (Appendix 1–Appendix 2).

#### 2.3.2. Health-Related Quality of Life Assessment

We used the QLQ-C30 questionnaire, elaborated by the European organization of research and treatment of cancer [14–19]. The questionnaire included thirty forced-choice questions (Appendix 3–Appendix 4), classified as follows:

- Symptom items (9 items, 13 questions): fatigue, nausea and vomiting, pain, dyspnea, insomnia, appetite loss, constipation, diarrhea, and financial difficulties
- Functional items (5 items, 15 questions): physical functioning, role functioning, emotional functioning, cognitive functioning, and social functioning
- Global health item (1 item, 2 questions)

Each question is answered by a score ranging from 1 to 4. For every item, a score is calculated according to answers, ranging from 0 to 100%. Mean scores of symptom items, functional items, and global health items are, then, obtained. We used an Arabic Tunisian dialectal version of QLQ-C30 which is widely used in Tunisia but still not validated [18].

### 2.4. Ethical Aspect

In their inclusion, patients were asked to sign a written consent. The study protocol was discussed then accepted on November 9th 2018, by the ethics committee of Abderrahmen Mami (study protocol No 10/2018).

### 2.5. Statistical Analysis

We performed initially a descriptive statistical analysis to get frequencies, percentages, and mean sociodemographic parameters. Quantitative and objective assessment of sleep quality and quality of life was provided. Then, a comparative statistical analysis was conducted to identify correlations between mean scores of questionnaires or percentages (Student *t*-test or nonparametric tests) and between categorical parameters or frequencies (Qui square test).

## 3. Results

We enrolled 107 patients during the study. Exclusion criteria were decisive in 53 cases. We kept 64 cases in the data collecting step. The mean age of our patients was 62.9 ± 8.18 years [42-85]. All patients were active smokers. Current smokers represented 48.4% of all patients. Most frequent past medical history were hypertension (23%) and COPD (21%), as mentioned in Table 1. Eastern Cooperative Oncology Group Performance Status (ECOG PS) in the 1st consultation was less than 2 in 62.5% of all patients.

From the 1st consultation to the obtaining of histological confirmation, the mean delay was 40.6 days. Using the TNM 9th edition, 59.4% of patients were in stage IV of the disease (Table 1). Four cycles of palliative chemotherapy was administered for all stage IV patients (59%). Curative concomitant chemoradiation was performed in 3 patients (4.7%). Chemotherapy associated to sequential radiotherapy was administered for 11 patients (17.2%). Curative chemotherapy without additional thoracic radiotherapy was administered for 12 patients (18.7%).

### 3.1. Sleep Quality–PSQI Questionnaire Analysis

**Figure 1:**
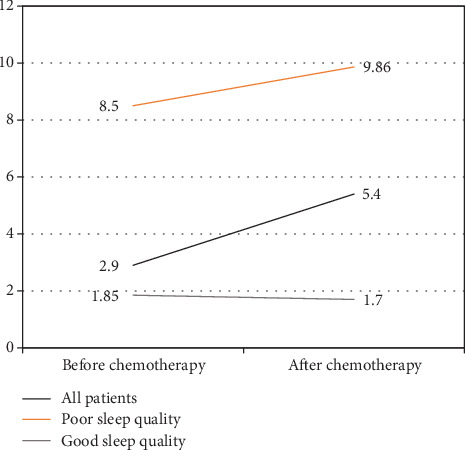
Mean Pittsburgh Sleep Quality Index (PSQI) score at baseline and after chemotherapy.

Ten patients (15.6% of all patients) had poor sleep quality before chemotherapy. After chemotherapy, 29 patients (45.3%) had poor sleep quality (Figure 1). PSQI score of all patients went from 2.9 before treatment to 5.4 after treatment (*p* = 0.001). Most reported sleep disturbances were daytime sleepiness, prolonged sleep latency, and nocturnal arousals (Table 2).

### 3.2. QLQ-C30 Questionnaire Analysis

High scored symptom items before chemotherapy onset were dyspnea (45.7%), anorexia (31.2%), and financial difficulties (42.7%). These findings showed the intensity of these complaints in our patients. Statistically significant rise after chemotherapy was noted in nausea and insomnia mean coefficients which reflected a worsening in these complaints (Table 3). Mean coefficient of symptom items of all patients was 22% before chemotherapy onset, then became 25.4% after chemotherapy. The coefficient was significantly less in patients with PSQI score < 5 and Performance Status score < 2.

Performance item mean scores before and after chemotherapy were nearly similar, between 64.2% and 78.6%. Statistically significant decrease after chemotherapy was mentioned in “physical functioning” and “social functioning” (Table 3). Mean coefficient of performance items of all patients was 71.3% before chemotherapy onset, then became 55.3% after chemotherapy. The coefficient was significantly better in patients with PSQI score < 5 and Performance Status score < 2. There was a statistically significant fall after chemotherapy in the mean coefficient of performance items of patients with preserved Performance Status score (Table 4).

### 3.3. Predictive Factors of Poor Sleep Quality: (Table 5)

Patients with good sleep quality and patients with bad sleep quality had a mean age of 62.9 years both. Clinical and demographic factors had no statistically significant effect on sleep quality in our patients (age, rural origin, histological subtype, disease stage, metastases, and their location).

Patients with poor sleep quality had a mean delay between their 1st consultation and histological confirmation significantly longer than patients with good sleep quality (47.2 days vs. 32.5 days; *p* = 0.05). As well, patients with poor sleep quality had a mean delay between histological confirmation and treatment onset significantly longer than patients with good sleep quality (28.1 days vs. 15.7 days; *p* = 0.0001).

Anxiodepressive mood was more frequently noted in poor sleep quality patients. Correlation between sleep quality and mood disturbances was statistically confirmed (*p* = 0.001). Factors related to treatment procedure (molecules of chemotherapy used on the first line, number of chemotherapy courses, in-patient or outpatient administration of treatment, and complication's occurring or severity) had no effect on sleep quality in our patients. Statistical multivariate analysis has shown an independent and significant correlation between sleep quality and therapeutic management delays (Table 6).

## 4. Discussion

Several key points distinguished our study and made its strength. The objective to identify predictive factors of sleep quality in cancer patients, its impact on quality of life, and its correlation with disease prognosis was reported by several studies [4, 6, 20, 21]. The correlation between quality of life and intensity of disease symptoms was previously established [20, 22]. Several studies also described a noticeable and sometimes unique correlation between quality of life and pain intensity. Strong recommendations derived from these studies to initiate early measurements to preserve quality of life and to control pain in cancer patients. Our methodology was to exclude patients with great pain levels, patients with poor general health, and patients on specific treatment interacting with sleep quality. That allowed us to select a coherent cohort of patients to evaluate the quality of life and its correlation with sleep quality independently from the influence of pain or general health [5]. Questionnaires are a validated tool to assess global health or symptom severity [12, 13]. Lack of objectivity for the PSQI questionnaire was noticed in several trials, especially in specific subgroups like cancer patients [23]. So, some authors used to combine the questionnaire to sleep diaries, actigraphy, or polysomnography [24–26]. There is a shortage of studies dedicated to analyse sleep quality in lung or chest cancer patients generally. However, sleep quality in many other types and localizations of cancer was assessed (Table 7, Table 8). Particular attention was granted to advanced stages, symptomatic patients, and survivor patients [20, 22, 27, 28]. These studies noted high PSQI scores. The blunt impact of the sociodemographic factors, the remarkable correlation between good sleep quality, and quality of life suggest an outstanding impact of the disease and the anxiodepressive reaction coming with it. A damaged “body image” could explain this fact, even with neither pain nor worsening of performance status. Poor quality of life in gynecologic cancer was widely described, even in operated patients (Table 8).

After cancer treatment, the prevalence of poor sleep quality was usually between 54% and 79%. Results from the study of Ilhan et al. [29] illustrated the influence of therapeutic methods (surgery, chemotherapy, and radiation therapy) on sleep quality in endometrium cancer patients: the PSQI mean score moved from 4.6 before treatment to 8.1 after treatment and poor sleep quality prevalence rised from 28% to 79%.

The effect of therapeutic procedure in sleep quality of cancer patients was randomly noted in several studies (Table 8). This fact could be explained by the heterogeneity in objectives or in the inclusion criteria of various trials. However, other findings must be underlined: Utility of questionnaires as assessment tools in cancer patients, high prevalence of sleep disturbances in cancer patients, and persistence of these troubles after specific treatment or even in survivor patients after a curative procedure. In lung cancer patients, sleep disturbances could be a “late-onset” phenomenon, noted mainly after chemotherapy. The relationship between quality of life and sleep disturbances does not make doubt. It seems to be strengthened when it is associated to mood disturbances. Also, anxiodepressive humor was described as a predictive factor of a declined quality of life [30–32]. Authors suggested to screen for poor quality of life and for mood disturbances, then to set up a psycho-oncological support program managed by the same structures of treatment. Such a nonpharmacological treatment could be effective in a cluster of poor sleeping patients.

## 5. Conclusions

Interactions between sleep quality, sleep composition, immune system, homeostasis, and endocrine functions are mutual and complicated. So, sleep quality could have an indirect impact on psychological equilibrium, response to treatment disease progression, and prognosis. Preservation of sleep quality is a major palliative care increasingly mentioned in advanced cancer management. Nevertheless, sleep quality deterioration before and after chemotherapy of lung cancer wasn't studied. Assessment of sleep quality is usually forgotten by physicians. There is, till today, no clear recommendations for the treatment of insomnia. Our study described a statistically significant relation between sleep quality, quality of mood and quality of life before, and after chemotherapy in locally advanced NSCLC patients. Further prospective studies are still needed to establish evidence-based recommendations for the treatment of sleep disturbances in cancer patients.

## Figures and Tables

**Table 1:** Clinical characteristics of the lung cancer patients.

	Number	%
Past medical history		
COPD	21	32.8
Pulmonary tuberculosis	10	15.6
Hypertension	23	35.9
Diabetes mellitus	9	14.1
Gastroduodenal ulcer	10	15.6
Benign prostatic hyperplasia	8	12.5
Performance status (PS)		
0	17	26.6
1	23	35.9
2	22	34.4
3	2	3.1
Stage		
IIIa	4	6.3
IIIb	13	20.3
IIIc	9	14.1
IVa	10	15.6
IVb	14	21.9
IVc	14	21.9
Delays (days)	Mean	Median
1^st^ X Ray–1^st^ consultation	18.5	17
1^st^ consultation–diagnosis	40.6	32

**Table 2:** Number and percent of sleep disturbances in poor sleep quality patients before and after chemotherapy.

Sleep disturbances	Baseline (*n* = 10)		After chemotherapy (*n* = 29)		*p*
Sleep hours < 6 hours	2 patients	20%	12 patients	41.4%	0.06
Sleep hours > 9 hours	4 patients	40%	5 patients	17.2%	0.09
Daytime sleepiness	7 patients	70%	26 patients	89.6%	0.14
Prolonged sleep latency	6 patients	60%	20 patients	68.9%	0.33
Delayed sleep phase disorder	0	0	1 patient	3.4%	0.18
Advanced sleep phase disorder	1 patient	10%	0	0	0.66
Nocturnal arousals	10 patients	100%	29 patients	100%	0.29

**Table 3:** Outcome of the mean scores of QLQ-C30 items after chemotherapy.

	Before chemotherapy		After chemotherapy		*p*
	Mean	Median	Mean	Median	
Items/symptoms					
Fatigue	32.3	33.3	34.9	33.3	0.61
Nausea	3.5	0	15.4	0	0.04
Pain	21.4	0	13	0	0.16
Dyspnea	45.7	33.3	41.7	33.3	0.37
Insomnia	13	0	22.9	0	0.05
Anorexia	31.2	0	41.2	66.7	0.08
Constipation	10	0	11	0	0.43
Diarrhea	0	0	0.5	0	0.10
Financial difficulties	42.7	33.3	44.8	33.3	0.52
Mean coefficient of symptom items	22	20.7	25.4	26.8	0.47
Performance items					
Physical functioning	64.2	66.7	12.7	13.3	0.001
Role functioning	76.6	83.3	73	83	0.47
Emotional functioning	71	66.7	65.7	66.7	0.21
Cognitive functioning	78.6	83.3	69	75	0.09
Social functioning	68	72.7	56	50	0.05
Mean coefficient of performance items	71.3	72.5	55.3	58.5	0.09

**Table 4:** Outcome of the mean coefficients before then after chemotherapy.

		Before chemotherapy		After chemotherapy		*p*
		Mean	Median	Mean	Median	
Mean coefficient of symptom items						
All patients (*n* = 64)		22	20.7	25.4	26.8	0.31
Performance status	0	12.4	11.1	17.5	12.3	0.14
	1	21.5	22.2	24.2	26.5	0.19
	2	29.4	30.8	32.5	31.5	0.30
	3	27.1	27	25.9	25.9	0.36
PSQI score	>5 (*n* = 29)	20.2	18.5	18.6	19.1	0.55
	≤5 (*n* = 35)	31.8	33.3	33.5	32.1	0.34
BMI	≤18 (*n* = 6)	29.9	33.3	31.4	33	0.42
	>18 (*n* = 58)	20.7	18.5	24.7	26.5	0.18
TNM stage	III (*n* = 26)	20	19	18.5	20.3	0.39
	IV (*n* = 38)	26	26	29.4	30.6	0.28
Mean coefficient of performance items						
Tous les patients (*n* = 64)		71.3	72.5	55.3	58.5	0.08
PS	0	79.1	86.6	62.5	70.7	**0.038**
	1	76.8	79	60.3	65.4	0.13
	2	62.7	61.8	46	45	0.11
	3	37.5	37.5	40.9	40.9	0.23
PSQI score	>5 (*n* = 29)	50.5	49	46	41.3	0.16
	≤5 (*n* = 35)	75.2	78.7	63	67	0.09

**Table 5:** Predictive factors of sleep quality–univariate statistical analysis.

		Good sleepers	Poor sleepers	*p*
Mean delays				
1^st^ consultation -histological confirmation (days)		47.2	32.5	0.05
Histological confirmation–treatment onset (days)		28.1	15.7	0.0001
Disease-related parameters				
Stage	III (%)	41.4	40	0.80
	IV (%)	58.6	60	
Histology	Adenocarcinoma (%)	58.6	54.3	0.57
	Squamous cell carcinoma (%)	27.6	34.3	
	Nonsmall cell carcinoma (%)	13.8	11.4	
Metastasis	Yes (%)	58.6	57.2	0.55
	No (%)	41.4	42.8	
Cerebral metastasis	Yes (%)	17.2	14.3	0.64
	No (%)	82.8	85.7	
Treatment-related parameters				
Chemotherapy course rate	One day per cycle (%)	13.8	17.2	0.49
	Two days per cycle (%)	86.2	82.8	
Hospitalisation during course	Inpatient (%)	34.5	31.4	0.71
	Outpatient (%)	65.5	68.6	
Grade IV complication	Yes (%)	20.7	37	0.09
	No (%)	79.3	63	
Sleep quality	Humor quality	Number of patients	%	*p*
PSQI > 5	Normal	3 patients	10.4%	0.001
	Depression	17 patients	58.6%	
	Anxiety	9 patients	31%	
PSQI ≤ 5	Normal	28 patients	80%	0.001
	Depression	5 patients	14.3%	
	Anxiety	2 patients	5.7%	

**Table 6:** Predictive factors of sleep quality–multivariate statistical analysis.

Variable	Univariate analysis	Multivariate analysis		
	*p*	Odds ratio adjusted	IC = 95% for Exp (B)	*p*
Mean delay to histological confirmation	0.05	0.89	0.83–0.96	0.004
Mean delay to treatment onset	0.0001	1.37	1.12–1.67	0.002
Chemotherapy complications	0.09	1.79	0.46–6.95	0.39
Depressive humor	0.001	1.60	0.59–4.34	0.35
Anxious humor	0.001	3.2	0.68–15.09	0.14

**Table 7:** Prevalence of insomnia in cancer patients.

Author	Prevalence
Delgado-Guay et al. [33]	85%
Beck et al. [34]	65%
Silberfarb et al. [28] lung cancer	50.3%
Silberfarb et al. [28] breast cancer	51%
Mansano-Schlosser and Ceolim [35]	47.4%
Our study: before chemotherapy	15.6%
Our study: after chemotherapy	45.3%

**Table 8:** Quality of life assessment and its correlation to sleep quality.

Author	Questionnaire	Type of cancer	Conclusion
Kerner et al. [36]	QLQ-C30 and ESAS	Breast or endometrium cancer	Enhancement of quality of life after CIM
Fortmann et al. [37]	HRQOL	All types of cancer/teenager patients	Correlation between quality of life, asthenia, and insomnia
Beck et al. [34]	MOS PCS et MOS MCS	Breast/stage I to IIIA	Correlation between quality of life, physical, and mental health
Trudel-Fitzgerald et al. [38]	Multidimensional fatigue inventory	Resected cancers of all types	Adjuvant treatment is correlated with more depression, anxiety, insomnia, asthenia, and pain
Armbruster et al. [39]	SF36	Endometrium/early stages	Correlation between mental health and sleep quality
Inhestern et al. [32]	SF8 et FAD-GF	All cancer types survivors	Several items (social and familial support) are correlated to anxiety and depression
Our study	QLQ-C30 and PSQI	Lung/advanced stages	Several quality of life items (nausea, insomnia, physical performance) worsened after chemotherapy

CIM: Complementary integrative medicine ESAS: Edmonton Symptom Assessment Scale; FAD-GF: general functioning scale of the Family Assessment Device; HR-QOL: Health related-Quality of life; MOS MCS: Medical Outcomes Study Mental Component Score; MOS PCS: Medical Outcomes Study Physical Component Score; SF36: Short form.

## Data Availability

The data used to support the findings of this study are available from the corresponding author upon request.

## Appendix

### A.1. Pittsburgh Sleep Quality Index (PSQI) Questionnaire in English Version.

Arabic dialectal version is available from the corresponding author upon request.
